# Spatio-Temporal Characteristics of the Supply and Demand Coupling Coordination of Elderly Care Service Resources in China

**DOI:** 10.3390/ijerph191610397

**Published:** 2022-08-20

**Authors:** Yijie Zhang, Mingli Zhang, Haiju Hu, Xiaolong He

**Affiliations:** 1 School of Economics and Management, Yanshan University, Qinhuangdao 066004, China; 2 Department of Public Education, Hebei University of Chinese Medicine, Shijiazhuang 050200, China; 3 School of Mechanical Engineering, Yanshan University, Qinhuangdao 066004, China

**Keywords:** elderly care service resources, supply and demand coupling coordination, BP neural network prediction, spatio-temporal characteristics

## Abstract

The current situation and future development of the supply and demand coupling coordination of elderly care service resources reflect the level of elderly care service resource allocation. Whether factors affecting its development can be found is the key to promote the accurate allocation of elderly care service. Based on the coupling coordination model, the supply and demand of elderly care service resources, the development circumstance and the spatio-temporal evolution of supply and demand coupling coordination are analyzed in this paper by using the data of the elderly care service resources in 31 regions and autonomous regions in China from 2010 to 2019. The result shows that there are regional differences in the development of supply and demand coupling coordination of elderly care service resources. The degree of supply and demand coupling coordination of elderly care service resources in the western and northern regions is lower than that in the eastern and southern regions. Although the level in most areas of supply and demand coupling coordination of elderly care service resources will improve in the future, there is still a gap from good coordination. In order to strengthen the supply of elderly care service resources, and promote the upgrade of the supply and demand of elderly care service resources, the government should start from the demand of the elderly to increase investment in infrastructure construction, investment in elderly care services resources, talent training and other aspects.

## 1. Introduction

The progress of medicine plays an extremely important role in the reproduction and development of human society as well as being a symbol of a country’s economic development level and social civilization level. According to the World Population Prospects (2019) released by the United Nations Department of Economic and Social Affairs, the number of people over the age of 65 has increased dramatically globally in recent years. Low fertility rates and longer life spans are aging populations in almost every country in the world. In 2020, the number of people aged 65 and above exceeded 700 million for the first time, reaching 723.484 million [1]. The number of people aged 65 and over is expected to reach 1.5 billion globally by 2050. According to the China Aging Research Report 2022, the number of elderly people aged 65 and above in China was 191 million in 2020, and is expected to reach 300 million in 2030 and 420 million in 2050 [2]. The elderly are high-risk groups of various kinds of chronic diseases. In China, 75% of the elderly suffer from more than one chronic disease. In addition, the one-child policy has led to the migration of workers to economically developed regions and city clusters, resulting in an increase in the number of empty nesters [3]. Without the support of traditional families, the elderly population’s demand for old-age services has further increased. However, the development of China’s old-age service system started late. China had to build an elderly care system basically from scratch in only a few decades.

Therefore, the Chinese government attaches great importance to the changing trend of population aging in China and has set an active response to population aging as a national strategy. Since 2015, the State Council, the Ministry of Civil Affairs and other relevant departments have continuously introduced a number of plans and measures to promote the rapid development of elderly care services. In major cities across the country, a elderly care service system pattern has been formed with home care as the main, community care as the auxiliary, and institutional care as the supplement. However, China has a large elderly population, and the imbalance between the supply and demand of elderly care services is widespread.

Coupling theory is an important method to study the interaction between two or more parallel systems. The supply and demand subsystems of the elderly care service resource system affect each other, and there is an interactive development relationship. Through the energy exchange between subsystems, it promotes the evolution of endowment service resource system from simple to complex, from low to high, from disorder to order. The allocation system of elderly care service resources also changes into a more advanced system in the process of coupling and coordination. In the supply of elderly care service resources, it is necessary to consider not only the rationalization of the layout of elderly care service facilities, but also the quantity and quality of elderly care service providers and managers, as well as the operating environment and legal guarantee suitable for the development of elderly care service. Meanwhile, the demand for elderly care service resources is affected by many factors such as the size of the elderly population and the consumption preference of the elderly. The expansion of demand quantity and cost of elderly care services will promote the development of elderly care service supply system. The optimization of elderly care service supply will improve service quality and accessibility, enhance service attractiveness, and further promote the match between the supply and demand of elderly care service resources.

The allocation of elderly care service resources is the key to the construction of elderly care service system, and the matching of the supply and demand of old-age service is an important symbol of improving the elderly care service system. Therefore, it is of great significance to deeply understand the coupling mechanism between the two systems and predict the coupling development trend of the system in the future to promote the matching of the supply and demand of elderly care resources. 

Elderly care service resources in different areas of the coupling between supply and demand coordination development is different, its development relationship can be discussed from two dimensions. On the one hand, the coupling degree of the supply and demand of elderly care service resources can be analyzed from the time dimension. To explore the difference, trend and volatility of the coupling and coordinated development of the supply and demand of each pension service resource. On the other hand, the spatial differences and evolution rules of the coupled and coordinated development of the supply and demand of elderly care service resources in different regions can be analyzed from the spatial dimension. Therefore, this study has the following detailed objectives: (1) Analyze the influencing factors of elderly care service resource demand and elderly care service supply. (2) Measure the coupling degree of the two and predict the future. (3) Analyze the spatial characteristics of supply and demand coupling level in different regions of China, and provide reference for the optimization of supply and demand matching.

## 2. Literature Review

**Figure 1:**
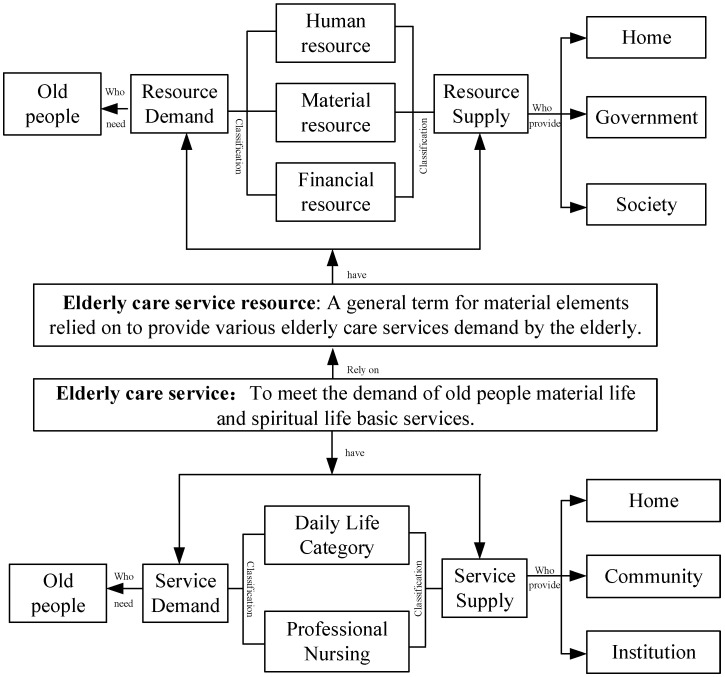
Schematic diagram of the relationship between the key concepts.

Elderly care service refers to the provision of necessary life services for the elderly, to meet their basic needs in material and spiritual life, it has certain instrumental properties [4]. Generally, elderly care services can be summarized as daily life care and professional nursing, including life care, nursing, education, culture and entertainment, legal advice and other content [5]. Based on the existing literature [6], this paper sorted out the related concepts of old-age service and old-age service resources. The schematic diagram of the relationship between the key concepts is shown in Figure 1.

The needs of the elderly are affected by many factors. There are differences in the needs of the elderly with different ages and residence conditions. The older the elderly are, the stronger their needs for social elderly care services are [7]. The physical health and economic status of the elderly are important factors affecting the elderly’s choice of pension mode [8]. The elderly have demand for hard housework and outdoor activities to help deal with personal affairs and leisure services [9]. In addition, children also have a certain impact on the elderly’s demand for care services, and the elderly’s satisfaction with their children’s support is inversely proportional to their willingness to stay in nursing homes [10]. Other studies have found that 20% of the elderly have needs for daily care, and 20% have needs for financial stewardess and other help [11]. Based on the research on the demand of nursing service for the aged, it is proposed that the country should establish an integrated health care service system from the aspects of supporting policy, manpower development, information and communication technology development [12].

Research on the supply of elderly care resources can be traced back to 1984, Evashwick et al. used the Anderson model to correlate service use with propensity, enablement, and need factors to analyze the reasons for health service use among 1317 older adults [13]. Since then, scholars have conducted in-depth studies on the influencing factors of elderly care service supply. By comparing the development of integrated care services and health and social care systems in England and the Netherlands, it is concluded that the key factors influencing their development include not only the socio-economic and political environment, but also the market of capital flow. Older persons, especially those in poor health, need a wide range of health social and residential care services to meet their health care needs, hence the need to develop an integrated care model [14]. Blanco (2013) proposed that to achieve effective management of long-term care service supply, it is necessary not only to clarify demand for elderly care services and nursing and health care goals, but also to cooperate and coordinate with government entities and provide financial support [15]. The development and construction of elderly care service facilities with the input of government and social funds will improve the elderly’s consumption willingness, further stimulate the consumption demand of the elderly, promote the increase in the income of pension institutions, promote the adjustment of elderly care service resource allocation structure, and achieve a virtuous cycle.

The previous literature has carried out a series of studies on the supply and demand of elderly care services, using qualitative methods to study the behavior pattern of the supply and demand side of elderly care services. The result have positive significance for health administrative agencies to implement action strategies [16]. Research on the gap between the supply and demand of elderly care services is helpful to improve the supply level of elderly care services and promote the match between supply and demand [17]. Coupling theory is an important theory to study the relationship between supply and demand, and studying the coupling between the two can provide methods to alleviate the contradiction between supply and demand [18]. Through the calculation of the coupling degree, the current situation of elderly care development can be analyzed, and further put forward policy suggestions to optimize the effective supply of elderly care services, improve the elderly care service environment and improve the quality of elderly care services [19]. The two subsystems of coupling coordination can interact and influence each other through energy exchange to form an interactive development relationship [20,21,22]. When two or more systems or elements are coupled, if the evolution direction of the two is consistent with the development direction of the system, and the system or elements promote each other and have a benign symbiosis, it is called positive coupling. If the evolution direction of the two is contrary to the development direction of the system, the system coupling will be unstable and imperfect, which will become the obstacle and resistance of the coupling operation. As a result, the system or elements will consume each other and decline, which is called negative coupling [23]. In addition, in the study of the matching of service supply and demand, the analysis of the spatial distribution of supply and demand is helpful to provide a reference for the rational planning of service resources [24,25,26]. Through spatial analysis, the correlation and difference of supply and demand related indicators in different regions can be obtained, and the reasons for these correlations and gaps can be further analyzed [27]. In spatial analysis, *Moran’s I* is often used to identify spatial agglomeration [28]. According to the results of spatial aggregation calculation, the regions with insufficient matching between supply and demand are determined [29].

To sum up, domestic and foreign scholars have more and more abundant researches on the matching of the supply and demand of elderly care service resources, but there is still room for improvement. At present, there is a lack of studies on the supply and demand of elderly care service resources based on coupling theory and spatial analysis. With the help of spatial autocorrelation analysis, the spatial variation rules of the coupling degree of elderly care service resources in different regions can be understood more clearly. Therefore, this paper regards the demand and supply of elderly care service resources as two parallel subsystems. From the perspective of the elderly service resource demand system, the elderly service resource demand promotes the development of the elderly service resource supply system through factors such as the elderly’s quantity, health status and consumption intention. From the perspective of the elderly care service resource supply system, the investment of government and social funds in the development and construction of elderly care service facilities and the improvement of elderly care service will improve the consumption willingness of the elderly, further stimulate the consumption demand of the elderly, promote the income increase in pension institutions, promote the adjustment of elderly care service resource allocation structure, and achieve a virtuous cycle.

## 3. Materials and Methods

### 3.1. Index System

The supply system of elderly care service resources is a complex system. There are two parallel and equivalent subsystems in the configure system of elderly care service resources: the demand and supply elderly care service resources, which influence and interact each other through exchanging energy in the operation of configuration of elderly care service resources. In this paper, the degree to which the two systems influence each other through their respective elements can be defined as the coupling coordination degree between the demand and supply in elderly care service, which reflects the degree of interaction between the two systems.

This paper selected the system indicators of supply and demand for elderly care service resources based on the principles of comprehensiveness, availability, measurability and comparability. Additionally, combined with the *Implementation Opinions of The State Council on Accelerating the Development of Elderly Care Service Industry*, the *Code for the Architectural Design of Elderly Care Facilities (GB50867-2013)* and other policy standards. The frequency statistical analysis method was used to select the indicators frequently used by researchers in recent years.

In terms of the supply of elderly care service resources, this paper divides them into human resources, material resources and financial resources. In the dimension of human resources for elderly care services, the service staff of elderly care institutions and community service centers are the main providers of elderly care services, and more workers represent more services for the elderly. Therefore, the number of employees in pension institutions at the end of the year (X_1_) and the number of employees in community service centers at the end of the year (X_2_) are selected to represent the supply level of human resources for old-age care services.

In terms of elderly care service human resources, the number of employees in pension institutions and community service centers are the main providers of elderly care service, and more employees means that the elderly can get more services.

In terms of the material resources of elderly care services, the larger the number of pension institutions, the wider the service area. The more pension beds, the more elderly inpatients can be admitted. The larger the building area of the pension institution, the more elderly people can be accommodated. Therefore, the number of nursing institutions (X_3_), nursing beds (X_4_), building area of nursing institutions (X_5_), the number of community nursing institutions (X_6_), and the number of community day care beds (X_7_) are selected to represent the supply level of material resources of elderly care services.

In terms of endowment service resources and financial resources, this paper mainly considers the government subsidies for the elderly in addition to pension, as well as financial support for pension institutions. The payments to the elderly include welfare payments such as the old-age allowance and the nursing allowance. In China, the government provides operating subsidies to pension institutions based on the number of the elderly in difficulty and the disabled who are admitted to the pension institutions. The elderly in difficulty refers to the elderly who have no ability to work, no source of livelihood, and no dependents or dependents. Therefore, three indicators are selected to represent the supply of financial resources for old-age services: the number of elderly people receiving old-age subsidy (X_8_), the welfare fund expenditure of the elderly (X_9_), and the subsidy level of old-age institutions (X_10_).

To sum up, there are three first-class indexes and ten second-class indexes in the elderly care service resource supply system, as is shown in Table 1:

In index selection of elderly care service resources demand, it is considered that the population aging degree and the level of economic development are different in all regions, and the elderly have different demands for elderly care services. This paper is based on comprehensive, availability, measurability and comparability principle, combined with the statistical items that can reflect the demand of elderly care service resources in the China Civil Affairs Statistical Yearbook, and determines the indexes of the elderly care service demand system.

The larger the number of elderly people, the greater the demand for elderly care services, and the greater the demand for elderly care service resources. Therefore, the size of the elderly population (Y_1_) is listed as among the indicators of the elderly service demand system. The old-age dependency ratio refers to the ratio between the number of elderly people and the number of working-age people in a country or region. The larger the dependency ratio, the more people the labor force will bear per capita, which means the more serious the burden of labor force, and the more elderly people need to seek elderly care services other than family members. So the dependency ratio of the elderly population (Y_2_) is selected as among the indicators to measure the scale of demand for elderly service resources. Per capita disposable income is generally used to measure the changes in a country’s living standard. The higher the per capita disposable income, the higher the purchasing power of elderly people for elderly care services, and the higher the demand for elderly care services resources. The number of pension institutions at the end of the year represents the actual demand scale of elderly care service resources, and the larger the number, the greater the demand for elderly care service resources. To sum up, the system indicators of old-age service demand are shown in Table 2 including the number of elderly and elderly dependency ratio, which represent the demand scale of elderly care service resources; per capita disposable income, which can reflect the price of demand for elderly care service resources; the number of the elderly in pension institutions at the end of the year, which can represent the actual scale of demand for elderly care service resources.

The indexes of resource demand for pension are shown in Table 2.

### 3.2. Data Collection

The index data mainly come from the *China Statistical Yearbook* and the *China Civil Affairs Statistical Yearbook*. The problem of population aging in China is shown—it has become increasingly prominent in the past decade. Therefore, the statistical data of 31 regions and municipalities in Mainland China from 2010 to 2019 are selected in this paper to analyze the coupling conditions between the supply and demand of elderly care service resources. Additionally, the data from 2020 to 2024 are forecasted through data in this decade. In addition, based on prediction calculation results of supply and demand coupling conditions of elderly care service resources from 2020 to 2024, the development of supply and demand coupling conditions of elderly care service resources in the next five years is analyzed.

A total of 4340 index data of the supply and demand system of elderly care service in 31 regions and municipalities from 2010 to 2019 are collected in this paper. Limited to the lack of space, actual data for this decade are not listed.

### 3.3. Methods

#### 3.3.1. BP Neural Network Time Series Prediction

Due to the different units and orders of magnitude of each index data, every index data should be standardized before calculation.

There are i evaluation indexes, j regions, and t evaluation years. Z represents the set of all data, and $Z_{ij}(t)$ represents the observed value of the i index in the t year in the j region (i=1,2,…,n;j=1,2,…,m), the expression is as follows:

(1) $$Z=(Z^{1},Z^{2},Z^{3},Z^{4},\cdots ,Z^{t}),\;Z^{t}=Z_{ij}(t)$$

Calculation of the i second-class index and the j region after standardizing can be expressed as

(2) $$Z_{ij}{\left(t\right)}^{\prime }=\left\{\begin{array}{l} \frac{Z_{ij}(t)-\min(Z_{1j}(t),Z_{2j}(t),\cdots ,Z_{nj}(t))}{\max(Z_{1j}(t),Z_{2j}(t),\cdots ,Z_{nj}(t))-\min(Z_{1j}(t),Z_{2j}(t),\cdots ,Z_{nj}(t))}(Positive\;indicators)\\ \frac{\max(Z_{1j}(t),Z_{2j}(t),\cdots ,Z_{nj}(t))-Z_{ij}(t)}{\max(Z_{1j}(t),Z_{2j}(t),\cdots ,Z_{nj}(t))-\min(Z_{1j}(t),Z_{2j}(t),\cdots ,Z_{nj}(t))}(Negative\;indicators) \end{array}\right.$$

In Equation (2), $Z_{ij}{}^{\prime }$ represents the value of the *i*th secondary index in the *j*th region after standardizing.

In order to analyze the evolution trend of the coupling conditions between the supply and demand of elderly care service resources in China, based on the existing data of each index in each region from 2010 to 2019, the ability of the BP neural network for time series forecasting is used to forecast the index data from 2020 to 2024 in this paper.

The BP neural network can approximate any nonlinear mapping by learning, and there are many methods for timing forecasting, such as the nonlinear auto-regressive model with exogenous inputs (NARX), the nonlinear auto-regressive (NAR) method, and the nonlinear input–output method. The nonlinear input–output method is suitable for time series forecasting with fewer forecast data and simple forecasting logic because of its fast convergence rate and reasonable solution accuracy. By observing the original sample data, of which the time series is stable, we can meet the applied requirements of the BP neural network. Therefore, this method is chosen for data forecasting in this paper. The calculation and test procedures and results are shown in Appendix A.

#### 3.3.2. The Entropy Weight Method

After summarizing the annual index data of elderly care service resources, the entropy method is used to determine the initial weight of each index in the supply and demand system of elderly care service resources. The entropy method determines the index weight by calculating the entropy of the observed value of each index. In information theory, entropy is used to measure the disorder degree of the system—the smaller the information entropy of the index, the greater the variation degree of the index and the higher the weight. Different from a subjective weighting approach, the entropy method can exclude the influence of subjective factors on index weight and objectively reflect the weight of every index. The specific steps are as follows:

First, $S_{ij}$, as the proportion of region j in index i, is calculated:

(3) $$S_{ij}=\frac{Z_{ij}{}^{\prime }}{\sum_{i=1}^{n}Z_{ij}{}^{\prime }}$$

$S_{ij}$ represents the proportion of region j in item i index in Equation (3).

According to the definition of information entropy in information theory, the calculated entropy:

(4) $$K_{j}=-\frac{1}{\ln n}\sum_{i=1}^{n}(S_{ij}\times \ln S_{ij})$$

$K_{j}$ represents the entropy in Equation (4).

The index difference coefficient equation can be expressed as:

(5) $$D_{j}=1-K_{j}$$

$D_{j}$ represents the index difference coefficient in Equation (5).

The index weight equation can be expressed as:

(6) $$W_{j}=\frac{D_{j}}{\sum_{j=1}^{n}D_{j}}$$

$W_{j}$ represents the index weight in Equation (6).

#### 3.3.3. The Coupling Coordination Model

The comprehensive evaluation of the elderly care service resource supply system and demand system are, respectively, calculated by the linear weighting method. The equation can be expressed as:

(7) $$T=\sum_{j=1}^{n}W_{j}Z_{ij}{}^{\prime }$$

In Equation (7), T represents the comprehensive evaluation score of the elderly care service resource supply system or demand system.

According to the coupling coordination model, the development level of coupling coordination among systems can be calculated as follows:

(8) $$C=\frac{2\sqrt{T_{1}\times T_{2}}}{T_{1}+T_{2}}$$

(9) $$D=\sqrt{C\times (\alpha T_{1}+\beta T_{2})}$$

In Equations (8) and (9), *C* represents the coupling degree of two systems, D represents the coupling coordination level of two systems, *α* represents the relative importance of the elderly care service resource supply system in the integration, and *β* represents the relative importance in the elderly care service resources demand system.

The increase in elderly care service resources demand will promote the elderly care service supply. However, the improvement of the elderly care service resource supply is also affected by other factors such as politics and economics. Therefore, the values of *α* and *β* were, respectively, set as 0.4 and 0.6 by expert interview. The value of coupling coordination conforms to 0≤D≤1—the closer D is to 1, the better the coupling coordination between systems, and vice versa.

Referring to previous research results [30,31], the coupling coordination level is divided into 10 categories and the coordination degree is divided into 3 categories. The coupling coordination of elderly care service resource supply and demand is poor coordination when the coupling degree is lower than 0.4. When the coupling degree is between 0.4 and 0.6, the coupling and coordination degree of elderly care resource supply and demand is medium coordination. When the coupling degree is greater than 0.6, this indicates that the current situation of the supply and demand coupling coordination of elderly care service resources is excellent coordination. The specific classification is shown in Table 3.

#### 3.3.4. The Spatial Autocorrelation Model

Spatial autocorrelation can effectively reflect the spatial dependence between the coupling coordination degree of the supply and demand of elderly care service resources in various regions. In this paper, *Moran’s I* index was used to calculate the spatial autocorrelation.

(10) $$Moran^{\prime }sI=\frac{\sum_{p=1}^{n}\sum_{q=1}^{n}W_{pq}(x_{p}-\bar{x})(x_{q}-\bar{x})}{\sum_{p=1}^{n}\sum_{q=1}^{n}W_{pq}}\cdot \frac{n}{\sum_{p=1}^{n}W_{pq}{\left(x_{p}-\bar{x}\right)}^{2}}$$

where $x_{p}$ and $x_{q}$ are the coupling coordination degrees of spatial unit p and q, $\bar{x}$ is the observed average, n is the number of study areas, and $W_{pq}$ is the spatial weight matrix. Generally, the value range of *Moran’s I* index is [−1, 1]. If the value of *Moran’s I* index is positive, the global spatial correlation is positive. If the value of *Moran’s I* index is negative, it is negatively correlated with space. If the value of *Moran’s I* index is 0, there is no spatial correlation.

## 4. Results

**Figure 2:**
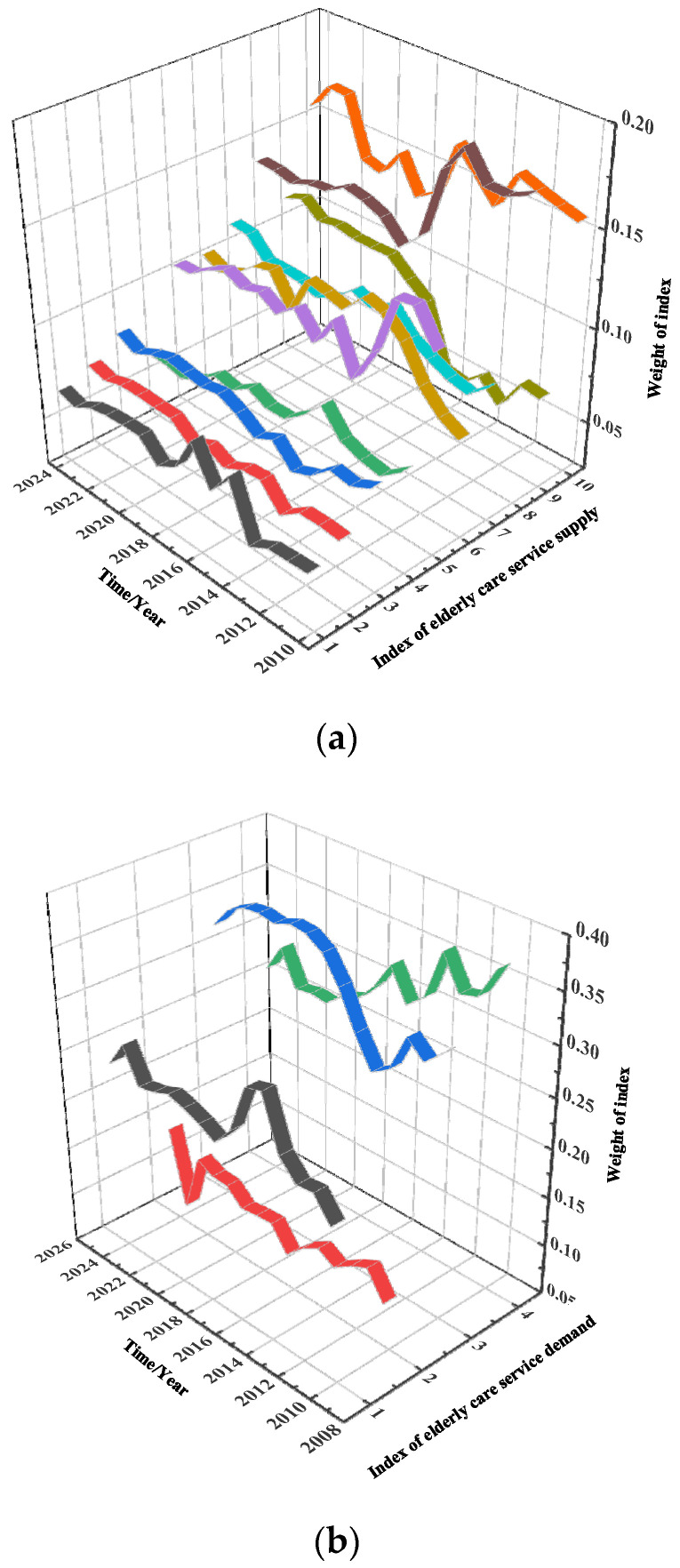
Supply and demand coupling coordination in each region from 2010 to 2019. (**a**) Supply index of elderly care service X. (**b**) Demand index of elderly care service Y.

MATLAB is used to calculate each index weights of the elderly care service resource supply system and demand system, based on actual data from 2010 to 2019 and forecast data from 2020 to 2024. The index weights are shown in Table 4 and Table 5 and in the Appendix B. The values of the same index in different years can represent the changing trend of the index weight, the values of different index in the same year can represent the importance of this index in that year. Graphically processing Table 4 and Table 5, the annual variation trend of the supply and demand index of elderly care service is shown in Figure 2.

As can be seen from Table 5, the variation trend of the coupling degree of 31 regions from 2010 to 2019.

Combining Table A1, Table A2 and Figure 2, the following conclusions can be obtained:

In the view of elderly care service supply index (X) changing trend, only the building area of pension institutions (X_5_), the number of community pension institutions (X_6_), the welfare expenditure of elderly people (X_9_) and the subsidy level of elderly service institutions (X_10_) have significantly fluctuate before 2015. Taking the building area of pension institutions (X_5_) as an example, its weight was 0.1302 in 2010, but dropped to 0.0946 in 2014, an there is a decline of 27.3%. After 2015, the weight coefficients of almost all index remain stable, and some indexes show a slight upward trend. The trend of elderly care service demand index (Y) is similar to supply indexes, showed a slight upward trend after 2015. Combined with the forecast data, this trend will continue in the next five years. The above phenomenon indicates that the coupling level between the supply and demand of elderly care service resources in China has been basically stable from 2015 to 2024 and has a trend of slow improvement.

In view of the value of index weight, the building area of pension institutions (X_5_), the number of elderly people receiving the old-age allowance (X_8_), the welfare expenditure of elderly people (X_9_) and the subsidy level of elderly service institutions (X_10_) are the highest, which are all greater than 0.1. The trend of forecast data shows that community pension is being taken seriously. The trend is also in line with the 2019 national active policy on community elderly care guidelines. As for the elderly care service demand index (Y), per capita disposable income (Y_3_) has the largest weight in 2019. The number of elderly in pension institutions at the end of the year (Y_4_) has the second one and elderly dependency ratio (Y_2_) has the lowest weight, while there is no violently changes in 2024. This indicates that the role of per capita disposable income and demand for pension institutions in models to be taken into key consideration.

According to Equations (7)–(9), MATLAB is used to calculate the coupling degree of 31 regions and its average value, and the results are shown in Table 4.

As can be seen from Table 4, the coupling degree of 31 regions and municipalities showed different variation trends from 2010 to 2019.

Based on Equations (7)–(9), MATLAB software is used to calculate the coupling degree and its average value of 31 regions from 2020 to 2024 based on the forecast data, and the results are shown in Table 5.

In order to make a spatial analysis of the matching between the supply and demand of elderly service resources in different regions, the *Moran’s I* index from 2010 to 2024 is calculated according to Equation (10), and the calculation results are shown in Table 6.

According to the data in Table 6, the values of *Moran’s I* index are all greater than 0, indicating that there is a certain positive correlation between the coupling coordination of the supply and demand of elderly care service resources in different regions in space. However, the small values indicate that the spatial correlation is not very strong, and there is no obvious pattern of variation with years.

## 5. Discussion

### 5.1. Analysis Based on Actual Data from 2010 to 2019

**Figure 3:**
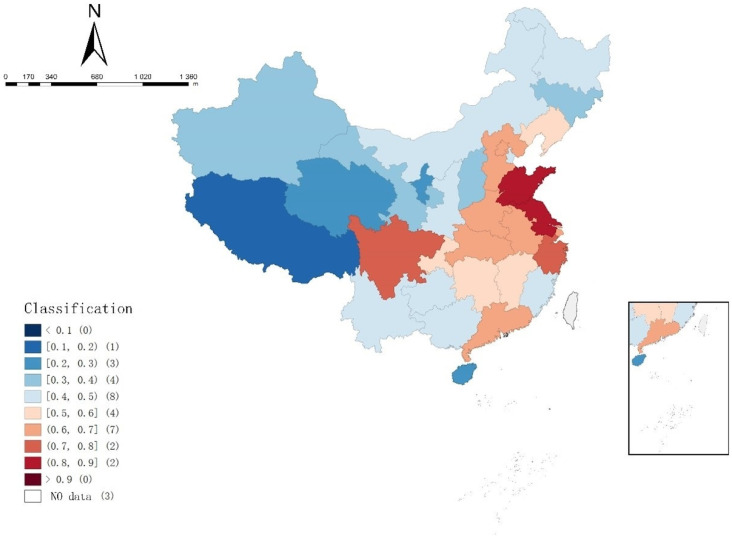
The spatial distribution of the coupling coordination degree of 31 regions (2010 to 2019).

The mean coupling degree of the supply and demand of elderly care service resources in each region in Table 6 were divided according to the coupling degree classification standard in Table 5, and expressed in the map by using Geoda software, as shown in Figure 3.

It can be seen from Table 5 and Figure 3 that the coupling degree of elderly care service resource supply and demand in Jiangsu and Shandong at good balance. Sichuan and Zhejiang at moderate balance, Beijing, Hebei, Shanghai, Anhui, Henan, Hubei, Guangdong, Liaoning, Jiangxi, Hunan and Chongqing are primary or barely balance. Meanwhile, there are seven regions at mild or moderate imbalance, eight regions at close to imbalance, and Xizang is still at serious imbalance. The coupling degree between the supply and demand of elderly care service resources in the western and northern regions is generally lower than that in the eastern and southern regions. There was a gradual upward trend from the west to the east. The LISA clustering map in the results of local *Moran’s I* index also showed that Shandong, Henan, Anhui, Jiangsu, Shanghai, Zhejiang and Jiangxi had a spatially high–high clustering trend. The reasons for this phenomenon may be as follows: the first is the influence of the size of the elderly population. According to the data, Shandong Province is the province with the largest elderly population in China, followed by Jiangsu, Sichuan and Henan Province. The larger the elderly population, the higher the demand for elderly care service, which stimulates the supply of elderly care service resources. Secondly, according to the level of economic development, Guangdong, Jiangsu, Shandong, Zhejiang and Henan rank the top five provinces in terms of GDP. A higher level of economic development means more financial resources can be invested in the construction of elderly care services, which is conducive to the supply of elderly care service resources. 

**Figure 4:**
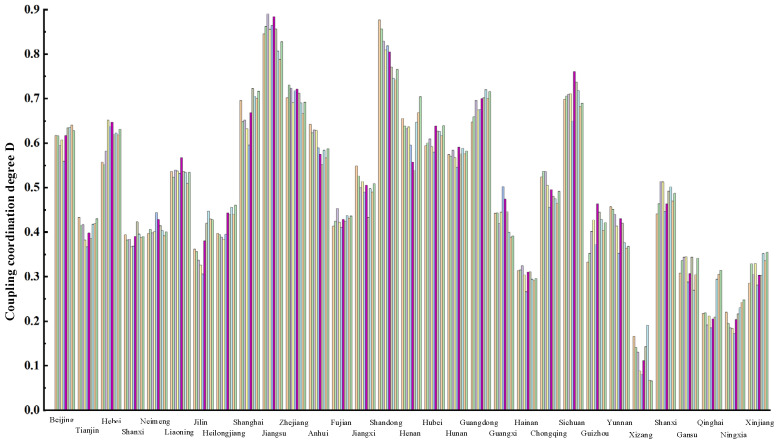
Coupling degree between supply and demand in every region from 2010 to 2019.

In order to represent the coupling degree of each region in different years more clearly, Table 6 is graphically represented as shown in Figure 4.

**Figure 5:**
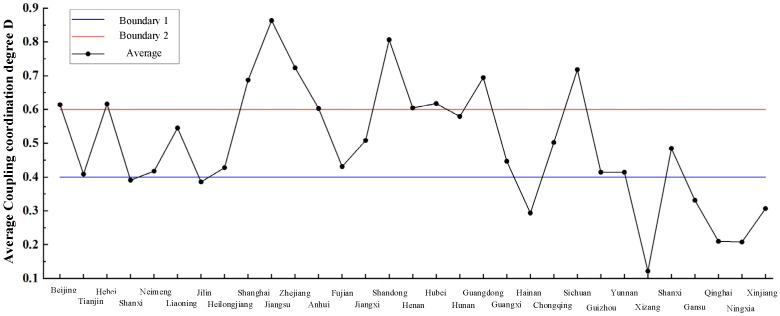
The mean coupling degree of supply and demand among various regions from 2010 to 2019.

As can be seen from Figure 4, the coupling degree between the supply and demand of elderly care service resources of each region and municipalities shows a regular variation over time. In Beijing, Hebei, Jilin, Heilongjiang, Shanghai, Guangdong and Guizhou, the coupling degree of elderly care service resource supply and demand shows an increasing trend. On the contrary, Anhui, Shandong, Guangxi and Yunnan shows a decreasing trend. The mean coupling degree of supply and demand among 31 regions in recent ten years is compared as shown in Figure 5.

Figure 5 shows two dividing lines. As can be seen from the figure, Shanghai, Jiangsu, Zhejiang, Shandong, Henan, Hubei, Guangdong and Sichuan have a high coupling coordination level in Hainan, Xizang, Gansu, Qinghai, Ningxia, and Xinjiang regions, the coupling coordination level of elderly care service resource supply and demand is poor. Therefore, the coupling degree between the supply and demand of elderly care service resources in most regions and cities in China needs to be further improved. The government and relevant departments need to increase investment in infrastructure construction, investment in elderly care services resources, talent training and other aspects. It is also shown from another perspective that the level of economic development has a certain impact on the coupling coordination degree—the higher the level of economic development, the higher the coupling coordination degree between the supply and demand of elderly care service resources.

### 5.2. Analysis Based on Forecast Data from 2020 to 2024

**Figure 6:**
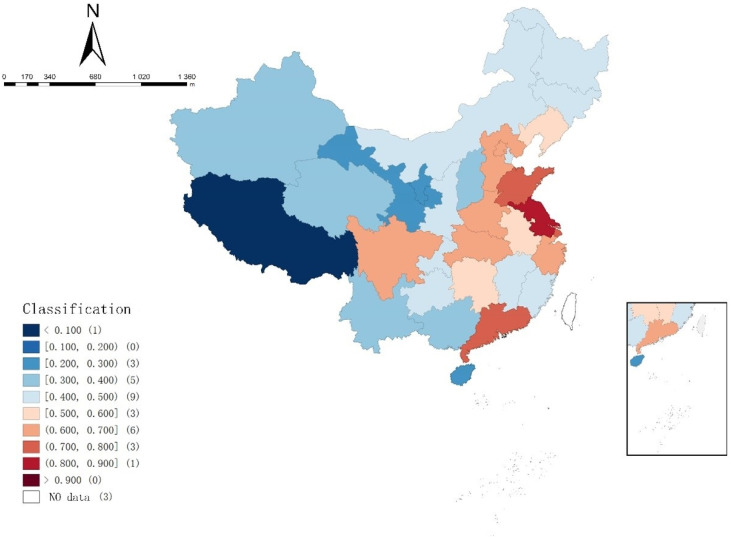
The spatial distribution of the coupling coordination degree of 31 regions (2020 to 2024).

The mean coupling degree between the supply and demand of elderly care service resources in each region in Table A1 were divided according to the coupling degree classification standard in Table 5, and expressed in the map by using Geoda software, as shown in Figure 6.

As can be seen from Figure 6, from 2020 to 2024, the average coupling degree of elderly care service resource supply and demand in 9 regions showed a downward trend, while only Guangdong, Qinghai and Liaoning showed an upward trend. It is urgent to introduce relevant policies to enhance the coupling degree between the supply and demand of elderly care service resources and improve the co-ordination of the supply and demand of elderly care service resources.

In terms of the value of coupling coordination degree, the predicted coupling coordination degree in 2020–2024 is worse than the actual coupling coordination degree. The reason for this phenomenon may be that the further growth of the elderly population leads to the continuous increase in pressure on the demand for elderly care services. If the supply policy of elderly care service resources is not adjusted in time to increase the supply, the imbalance between the supply and demand of elderly care services will increase further. The LISA clustering map in the local *Moran’s I* index results showed little change, only showing the phenomenon of high-low aggregation in Jiangxi and Guangdong. It can be observed from the changes of coupling coordination degree in different regions that the coupling coordination degree of Qinghai, Guangdong and Chongqing increased against the trend, but the reasons for the increase may be different. The increase in coupling coordination degree in Qinghai Province may be due to the fact that the size of the elderly population in this province is smaller than that in other provinces, and the dependency ratio of elderly population in Qinghai Province fluctuated from 2010 to 2019 and did not keep rising. Therefore, under the current policy of the supply and demand of elderly service resources, the coupling coordination degree in this province gradually increased. The main reason for the increase in the coupling coordination degree between Chongqing and Guangdong may be that the economic growth of the province is fast, which exceeds the growth trend of the demand for elderly care services. Therefore, it is not necessary to adjust the supply policy of elderly care services in a short period of time.

**Figure 7:**
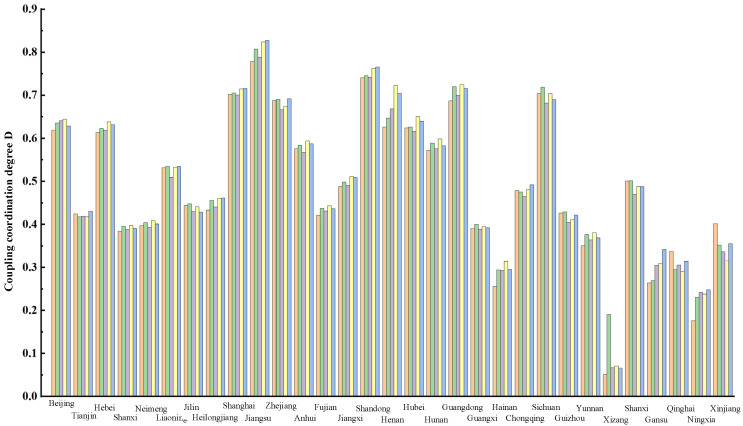
Coupling degree between supply and demand in every region from 2020 to 2024.

In order to represent the coupling degree of each region in different years more clearly, Table A1 is graphically represented as shown in Figure 7.

**Figure A2:**
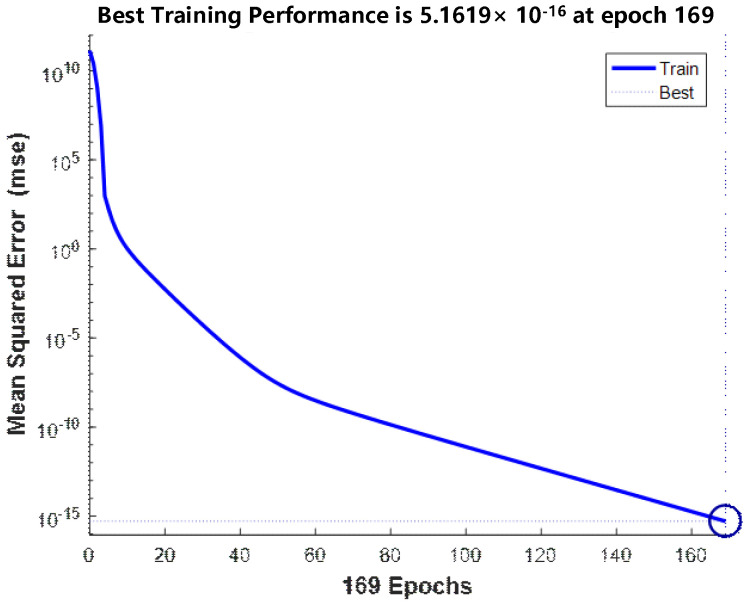
Performance of BP neural network training.

As can be seen from Figure A2, the coupling degree between the supply and demand of elderly care service resources in Hebei, Shanghai, Jiangsu, Henan, Guangdong, Gansu, Jiangxi and Fujian shows an obvious upward trend, which in other regions fluctuates slightly during the forecasting period, and it shows a slight upward trend in some regions. Compared with the classification of the coupling degree in Table 5, the coupling degree between the supply and demand of elderly care service resources in every region is similar to the result based on actual data. However, there is minimal change in some regions and municipalities.

**Figure 8:**
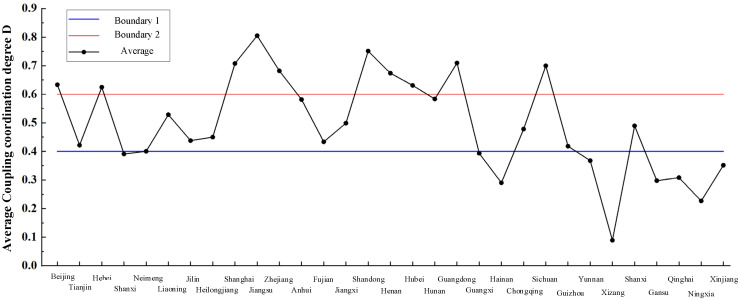
The mean degree of supply and demand coupling among various regions from 2020 to 2024.

Combined with Table 3 and Figure 8, it can be seen that the forecast results of the coupling degree between the supply and demand of elderly care service resources of all regions in China in the next five years are generally similar to the current situation from 2010 to 2019, Among 31 regions, Jilin, Guangxi, Yunnan three regions vary from medium to poor. Anhui vary from medium to good. It can be seen from the forecast data that the coupling degree between the supply and demand of elderly care service resources in most regions of China will remain in imbalance or barely balance state in the future, and the coupling coordination degree in the eastern region is higher than that in the western region.

## 6. Conclusions

Based on the coupling coordination model, this paper measures and predicts the coupling level of the supply and demand of elderly care resources in China. This paper analyzed the spatio-temporal evolution of supply and demand coupling coordination; revealed the matching, harmony, order and spatial characteristics of the development of the two systems; found the trend and cycle fluctuation of its development. This paper has enriched the theoretical understanding and practical research on the coupling coordination between the supply and demand of elderly care service resources.

The analysis results show that:

(1) Among all indicators, the building area of pension service institution, the number of elderly enjoying the old-age allowance, elderly welfare spending, the level of subsidy for ageing agencies, the number of community pension service institutions and the number of beds for community day care have a great influence on the coupling level of the supply and demand of elderly care service resources. The government can focus on improving the level of these indicators, so as to improve the coupling coordination level of elderly service resource supply and demand in this region.
(2) From the perspective of the coupling coordination degree of the supply and demand of the elderly service resources in each region, the coupling growth in each region is slow. In terms of spatial correlation, there is a weak positive correlation effect in each region, and the eight regions in the southeast are characterized by high–high aggregation in spatial autocorrelation.
(3) In most regions in China, the coupling degree between the supply and demand of pension service resources is barely imbalanced and close to imbalanced, and the regional differences are obvious. This shows that the coupling coordination degree of the supply and demand of elderly care service resources in the western and northern regions is lower than that in the eastern and southern regions.

According to the actual economic development level of each region and city, it is also shown from another perspective that the level of economic development has a certain impact on the supply and demand coupling coordination of elderly care service resources—the higher the level of economic development, the higher the coupling coordination degree between the supply and demand of elderly care service resources.

## Figures and Tables

**Table 1:** Indexes of elderly care service supply system.

Subsystem	First-Class Index	Second-Class Index	Unit
Elderly care service supply system X	Human resources	X_1_ Number of employees in pension institutions at the end of the year	pp
		X_2_ Number of community service center employees at the end of the year	pp
	Material resources	X_3_ Number of pension institutions	pcs
		X_4_ Number of beds for pension	pcs
		X_5_ Building area of pension institution	$m^{2}$
		X_6_ Number of community pension institutions	pcs
		X_7_ Number of community day care beds	pcs
	Financial resources	X_8_ Number of elderly receiving the old-age allowance	pp
		X_9_ Expenditure of welfare funds for the elderly	RMB Million Yuan
		X_10_ The level of subsidy provided by ageing agencies	yuan/pp·year

**Table 2:** Indexes of elderly care service demand system.

Subsystem	Index	Unit
Elderly care service demand system Y	Y_1_ Number of people aged over 65	pp
	Y_2_ elderly dependency ratio	%
	Y_3_ Disposable income	yuan
	Y_4_ Number of elderly in pension institutions at the end of the year	pp

**Table 3:** Classification standard of coupling coordination level.

Coupling Coordination Degree D	Coupling Coordination Level	Coordination Degree	Coupling Coordination Degree D	Coupling Coordination Level	Coordination Degree
[0, 0.1)	Extreme imbalance	Poor Coordination	[0.5, 0.6)	Barely balance	
[0.1, 0.2)	Serious imbalance		[0.6, 0.7)	Primary balance	Excellent Coordination
[0.2, 0.3)	Moderate imbalance		[0.7, 0.8)	Moderate balance	
[0.3, 0.4)	Mild imbalance		[0.8, 0.9)	Good balance	
[0.4, 0.5)	Close to imbalance	Medium Coordination	[0.9, 1)	Excellent balance	

**Table 4:** Coupling degree of 31 regions from 2010 to 2019 (actual data).

	2010	2011	2012	2013	2014	2015	2016	2017	2018	2019	Average
Beijing	0.6171	0.6162	0.5946	0.6075	0.5594	0.6172	0.6342	0.6312	0.6192	0.6455	0.6142
Tianjin	0.4328	0.4147	0.4171	0.3823	0.3677	0.3985	0.3857	0.3929	0.4367	0.4574	0.4086
Hebei	0.5573	0.5516	0.5824	0.6522	0.6368	0.6470	0.6189	0.6313	0.6437	0.6425	0.6164
Shanxi	0.3941	0.3823	0.3842	0.3675	0.3687	0.3908	0.4232	0.3838	0.4051	0.4090	0.3909
Inner Mongoria	0.3971	0.4063	0.3988	0.4011	0.4440	0.4289	0.4142	0.4365	0.4321	0.4171	0.4176
Liaoning	0.5365	0.5235	0.5397	0.5369	0.5314	0.5675	0.5363	0.5558	0.5684	0.5558	0.5452
Jilin	0.3621	0.3567	0.3374	0.3260	0.3059	0.3803	0.4199	0.4442	0.4562	0.4674	0.3856
Heilongjiang	0.3973	0.3945	0.3889	0.3834	0.3947	0.4436	0.4402	0.4764	0.4842	0.4763	0.4280
Shanghai	0.6962	0.6484	0.6519	0.6328	0.5963	0.6684	0.7225	0.7396	0.7514	0.7615	0.6869
Jiangsu	0.8455	0.8624	0.8904	0.8561	0.8643	0.8839	0.8568	0.8571	0.8678	0.8478	0.8632
Zhejiang	0.7020	0.7304	0.7231	0.6911	0.7175	0.7217	0.7128	0.7440	0.7576	0.7342	0.7234
Anhui	0.6421	0.6234	0.6298	0.6286	0.5901	0.5750	0.5520	0.5889	0.6011	0.5991	0.6030
Fujian	0.4133	0.4246	0.4532	0.4228	0.4109	0.4288	0.4248	0.4366	0.4438	0.4532	0.4312
Jiangxi	0.5487	0.5261	0.4997	0.5135	0.4901	0.5054	0.4325	0.5138	0.5240	0.5293	0.5083
Shandong	0.8767	0.8566	0.8292	0.8093	0.8188	0.8053	0.7711	0.7351	0.7822	0.7810	0.8065
Henan	0.6554	0.6386	0.6328	0.6361	0.5958	0.5576	0.5382	0.5509	0.5673	0.6744	0.6047
Hubei	0.5944	0.6003	0.6097	0.5921	0.5801	0.6390	0.6269	0.6297	0.6471	0.6571	0.6176
Hunan	0.5745	0.5713	0.5839	0.5675	0.5465	0.5911	0.5757	0.5836	0.5880	0.6126	0.5795
Guangdong	0.6478	0.6591	0.6960	0.6747	0.6753	0.7002	0.7022	0.7305	0.7331	0.7243	0.6943
Guangxi	0.4428	0.4431	0.4191	0.4447	0.5025	0.4746	0.4459	0.4430	0.4335	0.4178	0.4467
Hainan	0.3138	0.3149	0.3250	0.3038	0.2657	0.3102	0.3110	0.2712	0.2364	0.2855	0.2938
Chongqing	0.5240	0.5367	0.5362	0.5054	0.4556	0.4953	0.4805	0.4864	0.4937	0.5079	0.5022
Sichuan	0.6983	0.7056	0.7095	0.7110	0.6492	0.7612	0.7377	0.7404	0.7361	0.7299	0.7179
Guizhou	0.3331	0.3519	0.4020	0.4270	0.3720	0.4631	0.4448	0.4434	0.4634	0.4438	0.4145
Yunnan	0.4568	0.4513	0.4402	0.4134	0.3528	0.4305	0.4197	0.3942	0.3886	0.3950	0.4143
Tibet	0.1660	0.1412	0.1303	0.0884	0.0796	0.1115	0.1424	0.1306	0.1473	0.0789	0.1216
Shaanxi	0.4415	0.4638	0.5136	0.5126	0.4471	0.4632	0.4925	0.4938	0.5042	0.5155	0.4848
Gansu	0.3080	0.3366	0.3440	0.3445	0.2879	0.3064	0.3443	0.3512	0.3581	0.3315	0.3313
Qinghai	0.2170	0.2193	0.1912	0.2117	0.1851	0.2047	0.2095	0.2230	0.2136	0.2229	0.2098
Ningxia	0.2203	0.1951	0.1853	0.1837	0.1721	0.2034	0.2163	0.2420	0.2318	0.2279	0.2078
Xinjiang	0.2856	0.3294	0.3043	0.3299	0.2811	0.3029	0.3027	0.3026	0.3246	0.3034	0.3067

**Table 5:** Coupling degree of 31 regions from 2020 to 2024 (predicted data).

	2020	2021	2022	2023	2024	Average
Beijing	0.6186	0.6353	0.6407	0.6443	0.6281	0.6334
Tianjin	0.4243	0.4178	0.4189	0.4176	0.4302	0.4218
Hebei	0.6131	0.6224	0.6181	0.6383	0.6314	0.6247
Shanxi	0.3841	0.3954	0.3878	0.3973	0.3901	0.3909
Inner Mongoria	0.3968	0.4039	0.3926	0.4086	0.4006	0.4005
Liaoning	0.5317	0.5346	0.5096	0.5324	0.5347	0.5286
Jilin	0.4435	0.4476	0.4298	0.4408	0.4283	0.4380
Heilongjiang	0.4329	0.4559	0.4399	0.4602	0.4608	0.4499
Shanghai	0.7021	0.7049	0.7008	0.7145	0.7161	0.7077
Jiangsu	0.7783	0.8069	0.7885	0.8241	0.8272	0.8050
Zhejiang	0.6878	0.6906	0.6661	0.6741	0.6915	0.6820
Anhui	0.5757	0.5840	0.5670	0.5938	0.5870	0.5815
Fujian	0.4209	0.4369	0.4312	0.4429	0.4365	0.4337
Jiangxi	0.4871	0.4977	0.4905	0.5112	0.5088	0.4991
Shandong	0.7407	0.7456	0.7416	0.7625	0.7653	0.7511
Henan	0.6262	0.6468	0.6686	0.7229	0.7040	0.6737
Hubei	0.6239	0.6263	0.6162	0.6499	0.6392	0.6311
Hunan	0.5719	0.5886	0.5759	0.5987	0.5826	0.5835
Guangdong	0.6869	0.7199	0.7000	0.7246	0.7157	0.7094
Guangxi	0.3907	0.3999	0.3890	0.3949	0.3921	0.3933
Hainan	0.2560	0.2941	0.2920	0.3140	0.2956	0.2903
Chongqing	0.4785	0.4752	0.4649	0.4807	0.4917	0.4782
Sichuan	0.7036	0.7183	0.6820	0.7039	0.6900	0.6996
Guizhou	0.4261	0.4290	0.4044	0.4110	0.4213	0.4184
Yunnan	0.3501	0.3764	0.3641	0.3805	0.3683	0.3679
Tibet	0.0510	0.1905	0.0670	0.0710	0.0659	0.0891
Shaanxi	0.5007	0.5016	0.4701	0.4877	0.4872	0.4895
Gansu	0.2635	0.2693	0.3045	0.3087	0.3414	0.2975
Qinghai	0.3370	0.2944	0.3056	0.2909	0.3141	0.3084
Ningxia	0.1758	0.2305	0.2419	0.2377	0.2479	0.2268
Xinjiang	0.4015	0.3517	0.3361	0.3150	0.3548	0.3518

**Table 6:** *Moran’s I* index (2010–2024).

Year	2010	2011	2012	2013	2014
*Moran’s I*	0.186	0.201	0.146	0.143	0.153
Year	2015	2016	2017	2018	2019
*Moran’s I*	0.107	0.186	0.201	0.146	0.143
Year	2020	2021	2022	2023	2024
*Moran’s I*	0.153	0.107	0.186	0.201	0.146

**Figure A1:**
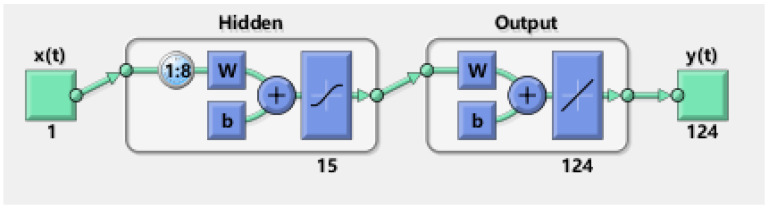
The structure of the BP neural network index forecast model of the elderly care service resources demand system.

**Figure A3:**
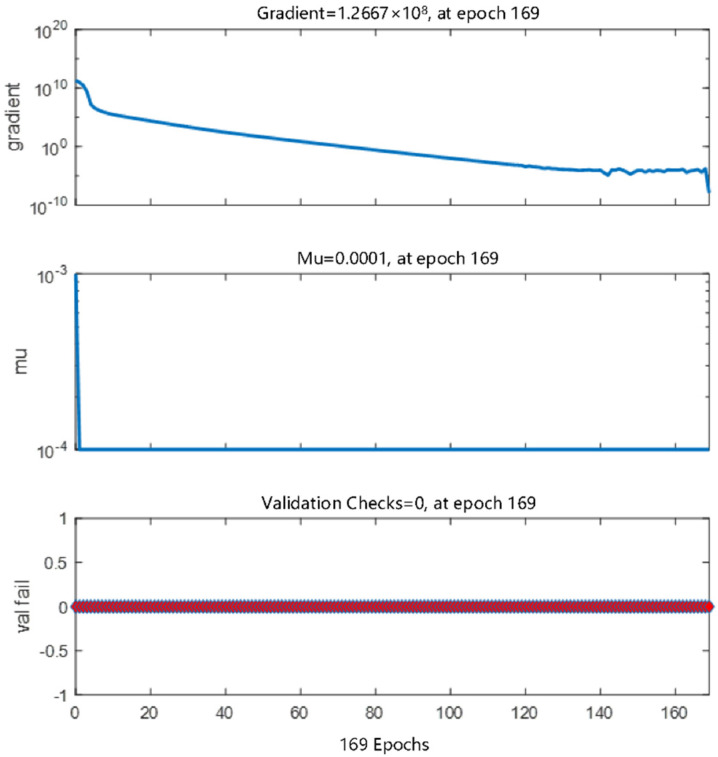
The training status diagram of the BP neural network.

**Figure A4:**
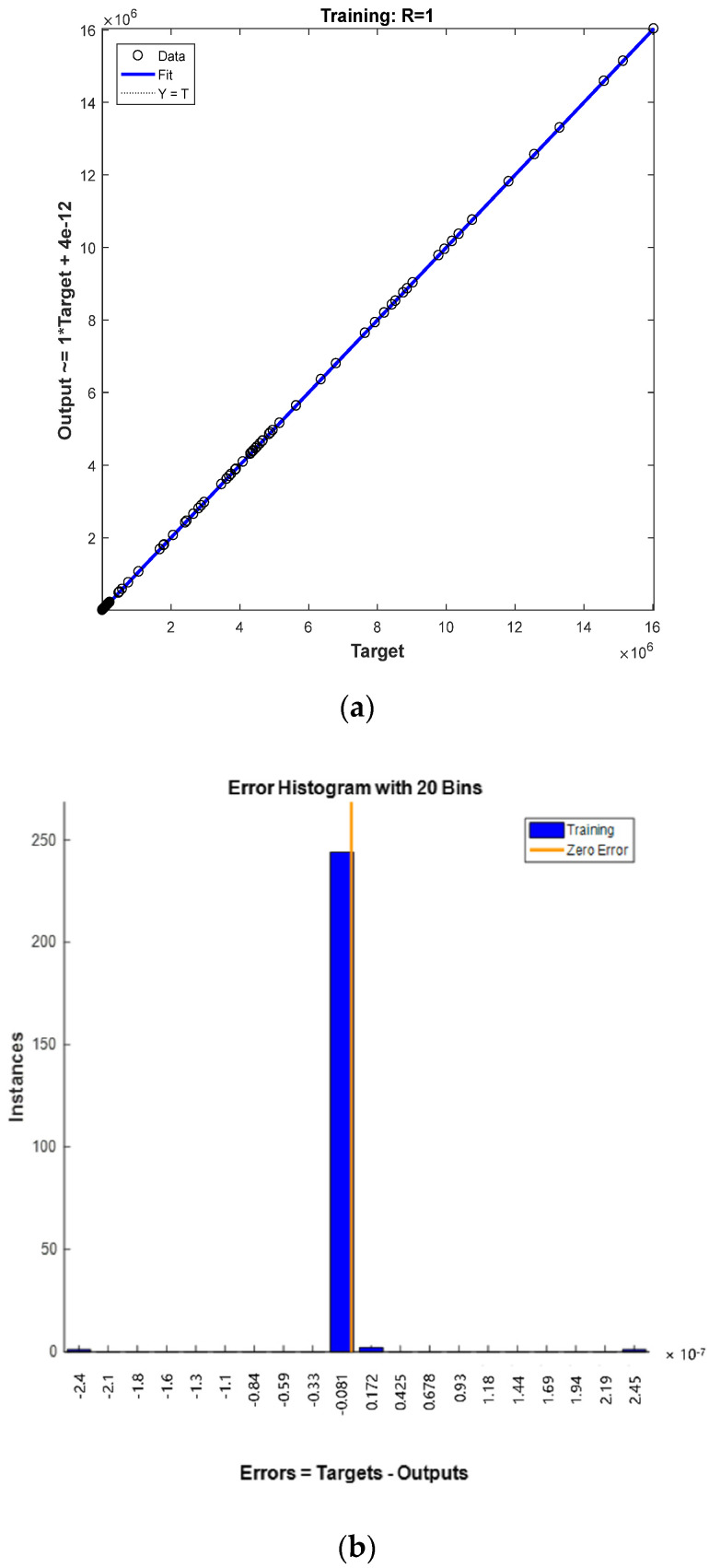
BP Neural network training regression and error histogram diagram. (**a**) Regression diagram. (**b**) Error histogram.

## Data Availability

Not applicable.

## Appendix A

In this paper, the BP neural network forecast adopts a three-layer structure, which consists of an input layer, a hidden layer and an output layer. Taking the forecast supply index as an example, the data from 2010 to 2019 are listed in a 10∗1 matrix as the input layer, and ten indexes in each of 31 provinces from 2010 to 2019 are normalized and listed in a 10∗124 matrix as the output layer by using Equations (1) and (2). In timing forecasting, the delay number is set to 8, indicating that the previous 8 timing values will be referred to every time the network fitting forecast is made. Kolmogorov’s theorem can be used to determine the number of hidden layer neurons in the range of 5–15, and the optimal number of hidden layer neurons is finally determined to be 15 through error comparison in a continuous experiment. The structure of the BP neural network index forecast model of the elderly care service resources demand system is finally determined, which is shown in Figure A1.

After determining the model, the BP neural network timing forecasting toolbox in MATLAB2020a software is used for network training. The training method is the Levenberg–Marquard method, which is a traditional and common method. In this method, the estimation parameter vector is linearly approximated in its neighborhood, and the derivative term above the second order is ignored, which is transformed into a linear least squares problem.

There are advantages such as fast convergence and this is suitable for time series forecasting. During training, 70% of the samples were used as training samples, 15% as validation samples, and the remaining 15% as test samples. After 169 training sessions, the network converges, and the training performance graph is shown in Figure A2.

As is shown in Figure A2, the final performance of the BP neural network meets the expected value after 169 rounds of training, and the final performance of the BP neural network satisfies the expected value after 169 training sessions—its mean-squared error (MSE) reaches $5.1619\times 10^{-16}$, and the performance of the network is satisfactory.

The training status diagram of the BP neural network is shown in Figure A3.

As is shown in Figure A3, after 169 rounds of training, the gradient of the BP neural network drops to $1.2667\times 10^{-8}$, the Mu is 0.0001 and it becomes 0 after validation checking, which shows that the performance of the BP neural network is satisfactory.

The conclusion can be reached from Figure A4a that the R value of the BP neural network in the regression analysis graph is 1, which represents a high matching degree of training samples, validation samples and test samples, as well as the high forecasting accuracy of the neural network.

In addition, it can be seen from Figure A4b that the errors of the BP neural network are significantly concentrated in the median, indicating that the neural network has high forecasting accuracy.

The forecast data are the data of 14 elderly care service supply and demand indexes in 31 provinces and municipalities from 2020 to 2024, with a total of 2170 data, which will not be listed in this section due to space limitations.

## Appendix B

**Table A1:** Index weight in the elderly care service resource supply/demand system from 2010 to 2019 (actual data).

	2010	2011	2012	2013	2014	2015	2016	2017	2018	2019
X_1_	0.0574	0.0568	0.0562	0.0506	0.0802	0.0665	0.0857	0.0661	0.0606	0.0704
X_2_	0.0642	0.0652	0.0649	0.0572	0.069	0.0681	0.063	0.0678	0.0632	0.0711
X_3_	0.081	0.0759	0.0776	0.0685	0.0643	0.0747	0.0699	0.0767	0.0812	0.0792
X_4_	0.0799	0.0718	0.0737	0.0775	0.09	0.0791	0.0714	0.0712	0.0759	0.0692
X_5_	0.1302	0.1471	0.1455	0.1137	0.0946	0.1196	0.1026	0.115	0.1069	0.113
X_6_	0.0785	0.084	0.0941	0.1112	0.1205	0.1233	0.112	0.1148	0.1167	0.0965
X_7_	0.0968	0.0876	0.0898	0.0946	0.0995	0.1106	0.1053	0.1094	0.1006	0.096
X_8_	0.1816	0.1766	0.1769	0.1914	0.1764	0.1396	0.1294	0.1398	0.1439	0.1459
X_9_	0.0744	0.0763	0.0593	0.0693	0.06	0.0618	0.0929	0.1002	0.1085	0.1081
X_10_	0.156	0.1589	0.1621	0.166	0.1454	0.1568	0.1678	0.139	0.1324	0.1507
Y_1_	0.2106	0.2332	0.2312	0.2493	0.3004	0.2958	0.2462	0.2262	0.2355	0.242
Y_2_	0.1021	0.1286	0.12	0.1083	0.1183	0.1081	0.0913	0.1145	0.1133	0.1111
Y_3_	0.3175	0.3011	0.3163	0.2779	0.2671	0.2961	0.332	0.3568	0.3673	0.3692
Y_4_	0.3697	0.3371	0.3324	0.3645	0.3141	0.3	0.3305	0.3025	0.2838	0.2778

**Table A2:** Index weight in the elderly care service resource supply/demand system from 2020 to 2024 (forecast data).

	2020	2021	2022	2023	2024
X_1_	0.0702	0.0695	0.0675	0.0622	0.0653
X_2_	0.0711	0.0716	0.0713	0.0685	0.0712
X_3_	0.0808	0.0826	0.0797	0.0752	0.0806
X_4_	0.0717	0.0637	0.0589	0.0612	0.0615
X_5_	0.1099	0.1146	0.1097	0.1031	0.1028
X_6_	0.1122	0.1094	0.101	0.0969	0.1015
X_7_	0.0993	0.1001	0.1001	0.1101	0.1112
X_8_	0.1398	0.1388	0.135	0.1375	0.1382
X_9_	0.1083	0.1103	0.1085	0.1161	0.1122
X_10_	0.1365	0.1394	0.1684	0.1691	0.1555
Y_1_	0.2469	0.2406	0.2373	0.2705	0.2445
Y_2_	0.1259	0.1267	0.1351	0.0757	0.15
Y_3_	0.3631	0.3663	0.3643	0.3559	0.3351
Y_4_	0.2642	0.2664	0.2633	0.298	0.2703
